# Awareness of Heart Attack Symptoms and Response Among Adults — United States, 2008, 2014, and 2017

**DOI:** 10.15585/mmwr.mm6805a2

**Published:** 2019-02-08

**Authors:** Jing Fang, Cecily Luncheon, Carma Ayala, Erika Odom, Fleetwood Loustalot

**Affiliations:** 1 Division for Heart Disease and Stroke Prevention, National Center for Chronic Disease Prevention and Health Promotion, CDC.

Heart disease is the leading cause of death in the United States (*1*). Heart attacks (also known as myocardial infarctions) occur when a portion of the heart muscle does not receive adequate blood flow, and they are major contributors to heart disease, with an estimated 750,000 occurring annually (*2*). Early intervention is critical for preventing mortality in the event of a heart attack (*3*). Identification of heart attack signs and symptoms by victims or bystanders, and taking immediate action by calling emergency services (9-1-1), are crucial to ensure timely receipt of emergency care and thereby improve the chance for survival (*4*). A recent report using National Health Interview Survey (NHIS) data from 2014 found that 47.2% of U.S. adults could state all five common heart attack symptoms (jaw, neck, or back discomfort; weakness or lightheadedness; chest discomfort; arm or shoulder discomfort; and shortness of breath) and knew to call 9-1-1 if someone had a heart attack (*5*). To assess changes in awareness and response to an apparent heart attack, CDC analyzed data from NHIS to report awareness of heart attack symptoms and calling 9-1-1 among U.S. adults in 2008, 2014, and 2017. The adjusted percentage of persons who knew all five common heart attack symptoms increased from 39.6% in 2008 to 50.0% in 2014 and to 50.2% in 2017. The adjusted percentage of adults who knew to call 9-1-1 if someone was having a heart attack increased from 91.8% in 2008 to 93.4% in 2014 and to 94.9% in 2017. Persistent disparities in awareness of heart attack symptoms were observed by demographic characteristics and cardiovascular risk group. Public health awareness initiatives and systematic integration of appropriate awareness and action in response to a perceived heart attack should be expanded across the health system continuum of care.

NHIS is an annual survey that collects health-related information on the civilian, noninstitutionalized U.S. population (*6*). In 2008, 2014, and 2017, the survey asked questions about symptoms of a heart attack and the best action to take when someone was thought to be having a heart attack. Five yes/no questions assessed whether the respondent was aware of these five symptoms of heart attack: 1) pain or discomfort in the jaw, neck or back; 2) feeling weak, lightheaded, or faint; 3) chest pain or discomfort; 4) pain or discomfort in the arms or shoulder; and 5) shortness of breath. Respondents were then asked, “If you thought someone was having a heart attack, what is the best thing to do right away?” The appropriate response was “Call 9-1-1” (or another emergency number). The total sample sizes were 21,781 (2008), 36,697 (2014), and 26,741 (2017). After excluding approximately 1% of respondents with missing information, the final analytic samples were 21,525 (2008), 36,289 (2014), and 26,480 (2017).

Descriptive characteristics of respondents included sex, age, race/ethnicity, and the highest level of education achieved. History of coronary heart disease (a condition caused by narrowing of the arteries that supply blood to the heart) included a reported history of coronary heart disease, myocardial infarction, or angina pectoris. Five selected self-reported cardiovascular disease (CVD) risk factors included hypertension (high blood pressure), high blood cholesterol, diabetes, current smoking, and obesity (body mass index ≥30 kg/m^2^ calculated from self-reported weight and height). Presence of CVD risk factors were evenly weighted and summed (0–5).

The prevalences of awareness of all five common heart attack symptoms, as well as the appropriate response when recognizing a heart attack (unadjusted and adjusted for age, sex, race/ethnicity, level of education, history of coronary heart disease, and number of CVD risk factors) were estimated overall and by selected demographic characteristics and CVD risk factors in 2008, 2014, and 2017. P-values for difference from 2008 to 2017 were obtained using the t-test; p-values <0.05 were considered statistically significant. Within-group comparisons for awareness and response were assessed by demographic characteristics and CVD risk factors in 2017 using the Wald F-test. SAS-callable SUDAAN (version 11.0, Research Triangle Institute) that accounted for the NHIS complex sample design, NHIS sampling weights, and design variables was used for all analyses.

During the three study years, the adjusted percentage of all survey respondents aware of the five common heart attack symptoms increased from 39.6% (2008), to 50.0% (2014), and to 50.2% (2017) (p-value for difference <0.001) (Table 1). Similar increases were observed in the unadjusted percentages and in all subgroups by demographic characteristics and CVD risk factors, except among those with four or five CVD risk factors. In 2017, knowledge of the five heart attack symptoms was lower among men, younger age groups, racial/ethnic minorities (especially non-Hispanic Asians and Hispanics), and persons with lower levels of educational attainment than among women, older adults, non-Hispanic whites, and adults with at least a high school education.

**TABLE 1:** Unadjusted and adjusted prevalence of knowledge of five common heart attack signs and symptoms by demographic characteristics, history of coronary heart disease (CHD), and number of cardiovascular disease (CVD) risk factors and change from 2008 to 2017 — National Health Interview Survey, United States, 2008, 2014, and 2017

Characteristic	Unadjusted					Adjusted*				
	2008	2014	2017	Percentage point change 2008 to 2017^†^	p-value (2008 versus 2017)	2008	2014	2017	Percentage point change 2008 to 2017^†^	p-value (2008 versus 2017)
	% (SE)	% (SE)	% (SE)			% (SE)	% (SE)	% (SE)		
**Total**	**39.4 (0.5)**	**49.9 (0.5)**	**50.4 (0.6)**	**10.9**	**<0.001**	**39.6 (0.5)**	**50.0 (0.5)**	**50.2 (0.5)**	**10.6**	**<0.001**
**Sex** ^§^										
Men	35.5 (0.7)	45.9 (0.6)	45.9 (0.7)	10.4	<0.001	35.7 (0.6)	45.9 (0.6)	45.6 (0.6)	9.8	<0.001
Women	43.1 (0.7)	53.7 (0.6)	54.5 (0.7)	11.4	<0.001	43.3 (0.7)	53.8 (0.6)	54.4 (0.6)	11.2	<0.001
**Age group (yrs)** ^§^										
18–44	32.5 (0.7)	41.8 (0.6)	43.5 (0.7)	11.0	<0.001	34.9 (0.7)	44.9 (0.7)	46.4 (0.7)	11.2	<0.001
45–64	46.7 (0.8)	57.3 (0.7)	55.6 (0.8)	8.9	<0.001	44.5 (0.8)	55.4 (0.7)	53.8 (0.8)	9.5	<0.001
≥65	45.3 (0.9)	56.7 (0.8)	57.4 (0.9)	12.1	<0.001	42.2 (0.9)	52.8 (0.8)	53.2 (0.9)	11.1	<0.001
**Race/Ethnicity** ^§,¶^										
White	45.2 (0.6)	55.8 (0.6)	56.6 (0.6)	11.4	<0.001	44.6 (0.6)	54.5 (0.6)	54.8 (0.6)	10.2	<0.001
Black	30.0 (1.1)	44.2 (1.1)	42.7 (1.4)	12.8	<0.001	31.2 (1.1)	44.9 (1.0)	43.1 (1.3)	11.6	<0.001
Asian	25.2 (2.0)	30.1 (2.0)	33.1 (2.6)	8.0	0.015	25.7 (1.9)	30.7 (2.0)	33.5 (2.5)	7.6	0.017
Hispanic	22.7 (0.9)	34.1 (1.1)	35.2 (1.3)	12.5	<0.001	27.0 (1.1)	38.9 (1.1)	38.9 (1.3)	11.1	<0.001
Other	30.9 (2.2)	43.8 (1.8)	47.0 (2.1)	16.1	<0.001	31.6 (2.2)	43.3 (1.8)	46.4 (2.1)	15.2	<0.001
**Education** ^§^										
Less than HS	28.3 (1.0)	39.3 (1.2)	40.5 (1.2)	12.1	<0.001	28.9 (1.0)	41.5 (1.2)	42.3 (1.2)	13.3	<0.001
HS graduate	39.5 (0.9)	49.2 (0.8)	47.9 (0.8)	8.5	<0.001	37.1 (0.8)	47.1 (0.8)	46.4 (0.8)	9.4	<0.001
Some college	44.9 (0.8)	55.8 (0.7)	54.2 (0.8)	9.3	<0.001	43.0 (0.8)	53.8 (0.7)	52.4 (0.8)	9.3	<0.001
College graduate	46.4 (1.0)	55.1 (0.7)	56.7 (0.8)	10.3	<0.001	45.5 (1.0)	54.0 (0.7)	56.0 (0.8)	10.5	<0.001
CHD**^,††^										
With CHD	51.9 (1.4)	61.9 (1.5)	59.2 (1.4)	7.3	<0.001	47.2 (1.4)	56.9 (1.5)	53.7 (1.4)	7.6	<0.001
Without CHD	38.6 (0.6)	49.2 (0.5)	49.8 (0.6)	11.2	<0.001	39.1 (0.5)	49.5 (0.5)	49.9 (0.5)	10.8	<0.001
**No. of CVD risk factors^§,§§^**										
0	35.0 (0.8)	46.4 (0.7)	45.9 (0.8)	11.0	<0.001	36.9 (0.8)	48.5 (0.7)	47.8 (0.8)	10.9	<0.001
1	38.1 (0.8)	49.2 (0.7)	49.8 (0.8)	11.7	<0.001	38.6 (0.8)	49.5 (0.7)	49.9 (0.7)	11.2	<0.001
2	45.0 (0.9)	52.8 (0.9)	55.2 (0.9)	10.1	<0.001	42.8 (0.9)	50.5 (0.9)	52.6 (0.9)	9.7	<0.001
3	46.7 (1.4)	57.2 (1.2)	57.8 (1.2)	11.1	<0.001	43.8 (1.3)	54.1 (1.2)	54.4 (1.2)	10.8	<0.001
4–5	51.0 (2.3)	57.8 (1.8)	56.0 (1.8)	5.0	0.091	47.9 (2.3)	55.0 (1.8)	52.8 (1.8)	5.0	0.124

**Abbreviations:** HS = high school; SE = standard error.

* Adjusted by age, sex, race/ethnicity, education, history of CHD, and CVD risk factor.

^†^ Estimates might differ from manual calculations because of rounding.

^§^ p<0.001 from Wald F of adjusted percentage by characteristics for 2017 data.

^¶^ Persons identified as Hispanic might be of any race. Persons identified as white, black, Asian, or other race are non-Hispanic.

** p<0.05 from Wald F of adjusted percentage by characteristics for 2017 data.

^††^ Includes self-reported coronary heart disease, angina pectoris, or myocardial infarction.

^§§^ Includes self-reported hypertension, high cholesterol, diabetes, smoking, or obesity.

The adjusted percentage of survey respondents who knew to call 9-1-1 in the event of a suspected heart attack increased across the observation period, from 91.8% (2008) to 93.4% (2014), and to 94.9% (2017) (p-value for difference <0.001) (Table 2). A significant increase in prevalence of knowing to call 9-1-1 was observed in all demographic subgroups and CVD risk factors, except among non-Hispanic Asians and respondents with history of coronary heart disease. In 2017, the adjusted percentage persons of appropriately responding to a heart attack by calling 9-1-1 was lower among men, adults aged ≥65 years, non-Hispanic Asians, persons with less than a high school education, and those having four or five CVD risk factors than among women, adults aged 18–44 years and 45–64 years, non-Hispanic whites and non-Hispanic blacks, persons with at least a high school diploma, and those with fewer than four CVD risk factors.

**TABLE 2:** Unadjusted and adjusted prevalence of knowing to call 9-1-1 in response to a heart attack, by demographic characteristics, history of coronary heart disease (CHD), and number of cardiovascular disease (CVD) risk factors, and change from 2008 to 2017 — National Health Interview Survey, United States, 2008, 2014, and 2017

Characteristic	Unadjusted					Adjusted*				
	2008	2014	2017	Percentage point change 2008 to 2017^†^	p-value (2008 versus 2017)	2008	2014	2017	Percentage point change 2008 to 2017^†^	p-value (2008 versus 2017)
	% (SE)	% (SE)	% (SE)			% (SE)	% (SE)	% (SE)		
**Total**	**91.9 (0.3)**	**93.3 (0.3)**	**94.8 (0.3)**	**3.0**	**<0.001**	**91.8 (0.3)**	**93.4 (0.3)**	**94.9 (0.2)**	**3.1**	**<0.001**
**Sex** ^§^										
Men	91.2 (0.4)	92.8 (0.4)	94.4 (0.3)	3.2	<0.001	91.1 (0.4)	92.8 (0.4)	94.4 (0.3)	3.4	<0.001
Women	92.5 (0.3)	93.8 (0.3)	95.2 (0.3)	3.0	<0.001	92.5 (0.3)	93.9(0.3)	95.3 (0.3)	2.8	<0.001
**Age group (yrs)** ^¶^										
18–44	92.8 (0.4)	94.4 (0.3)	95.8 (0.4)	3.0	<0.001	92.9 (0.4)	94.6 (0.3)	95.9 (0.4)	3.0	<0.001
45–64	92.1 (0.4)	93.1 (0.5)	94.6 (0.3)	2.5	<0.001	91.9 (0.4)	93.0 (0.5)	94.5 (0.3)	2.5	<0.001
≥65	88.4 (0.6)	91.1 (0.5)	92.9 (0.4)	4.4	<0.001	88.6 (0.6)	91.0 (0.5)	92.8 (0.4)	4.4	<0.001
Race/Ethnicity^¶,^**										
White	92.3 (0.3)	94.0 (0.3)	95.3 (0.2)	3.0	<0.001	92.3 (0.3)	94.0 (0.3)	95.4 (0.2)	3.1	<0.001
Black	91.8 (0.7)	93.8 (0.6)	95.0 (0.8)	3.2	0.002	91.8 (0.7)	93.7 (0.6)	95.0 (0.8)	3.3	0.002
Asian	89.8 (1.3)	87.9 (1.4)	91.2 (1.7)	1.4	0.519	89.2 (1.4)	87.1 (1.5)	90.8 (1.8)	1.6	0.462
Hispanic	90.5 (0.7)	91.3 (0.6)	93.6 (0.6)	3.1	<0.001	90.8 (0.7)	91.6 (0.6)	93.7 (0.6)	2.9	0.001
Other	89.4 (1.5)	93.6 (1.2)	93.7 (0.9)	4.3	0.013	88.6 (1.6)	93.1 (1.2)	93.2 (1.0)	4.5	0.011
**Education^¶^**										
Less than HS	87.7 (0.8)	89.5 (0.7)	92.3 (0.7)	4.6	<0.001	89.0 (0.7)	90.9 (0.6)	93.4 (0.6)	5.1	<0.001
HS graduate	91.7 (0.5)	93.2 (0.5)	94.4 (0.4)	2.7	<0.001	91.8 (0.5)	93.5 (0.5)	94.7 (0.4)	3.0	<0.001
Some college	92.6 (0.5)	93.5 (0.5)	95.2 (0.3)	2.6	<0.001	92.4 (0.5)	93.5 (0.5)	95.3 (0.3)	2.9	<0.001
College graduate	93.3 (0.4)	94.2 (0.4)	95.9 (0.3)	2.6	<0.001	93.1 (0.4)	94.2 (0.4)	95.9 (0.3)	2.8	<0.001
**CHD^§,††^**										
With CHD	90.8 (1.0)	90.2 (0.9)	91.7 (0.8)	0.9	0.454	92.7 (0.8)	92.3 (0.8)	93.5 (0.7)	1.0	0.436
Without CHD	91.9 (0.3)	93.5 (0.3)	95.0 (0.3)	3.1	<0.001	91.7 (0.3)	93.4 (0.3)	95.0 (0.3)	3.2	<0.001
**No. of CVD risk factors^§§^**										
0	92.1 (0.4)	94.0 (0.3)	95.4 (0.4)	3.3	<0.001	91.5 (0.4)	93.6 (0.3)	95.0 (0.5)	3.2	<0.001
1	92.0 (0.4)	94.0 (0.4)	95.1 (0.3)	3.1	<0.001	91.9 (0.4)	93.9 (0.4)	95.1 (0.3)	3.2	<0.001
2	92.0 (0.5)	92.2 (0.7)	94.5 (0.4)	2.5	<0.001	92.4 (0.5)	92.7 (0.7)	94.9 (0.4)	2.7	<0.001
3	91.4 (0.8)	91.9 (0.8)	93.6 (0.6)	2.2	0.028	92.3 (0.7)	92.9 (0.7)	94.4 (0.6)	2.4	0.017
4–5	87.6 (1.7)	91.3 (0.9)	91.8 (1.2)	4.2	0.042	89.1 (1.5)	92.5 (0.8)	93.1 (1.1)	4.6	0.028

**Abbreviations:** HS = high school; SE = standard error.

* Adjusted by age, sex, race/ethnicity, education, history of CHD, and CVD risk factor.

^†^ Estimates might differ from manual calculations because of rounding.

^§^ p<0.05 from Wald F of adjusted percentage by characteristics for 2017 data.

^¶^ p<0.0001 from Wald F of adjusted percentage by characteristics for 2017 data.

** Persons identified as Hispanic might be of any race. Persons identified as white, black, Asian, or other race are non-Hispanic.

^††^ Includes self-reported coronary heart disease, angina pectoris, or myocardial infarction.

^§§^ Includes self-reported hypertension, high cholesterol, diabetes, smoking, or obesity.

## Discussion

Delays in receipt of appropriate care lead to worse outcomes among heart attack victims (*3*). Although this nationally representative survey indicates improvement in the percentage of adults who know the signs and symptoms of a heart attack and to call 9-1-1 if they witness someone having a heart attack, in 2017, approximately half of respondents (50.2%) knew all five common heart attack signs and symptoms, and disparities in awareness and response exist among all demographic groups and by CVD risk status.

Data from 14 states reporting in the 2005 BRFSS found that 85.8% of respondents had the knowledge to call 9-1-1 as the first action when witnessing a heart attack and 31% were aware of all five heart attack symptoms (*4*). Although the percentage of persons with this knowledge was higher in this study than in the 2005 BRFSS, disparities by sex, race/ethnicity and level of education persisted. Both the BRFSS data and estimates from this report identify a need for increased awareness regarding the signs and symptoms of one of the most common important health events that can occur in persons in the United States. Recognizing this need, the U.S. Department of Health and Human Services *Healthy People 2020* (HP2020) program included objectives specifically calling for an increase in the awareness of heart attack signs and symptoms and the appropriate response (*7*). Using the 2008 NHIS data as the HP2020 baseline, the target for awareness of five common heart attack symptoms was set at 43.6% (10% increase from the 2008 adjusted prevalence of 39.6%), and the target for knowing to call 9-1-1 if someone is having a heart attack was set at 93.8% (2% increase from 91.8%). Although data from the current study indicate that in 2017 these goals for awareness of heart attack symptoms (50.2%) and calling 9-1-1 (94.9%) were met overall, estimates for certain subpopulations remained below the HP2020 target, including racial/ethnic minorities and adults with less than a high school education.

Many educational efforts have historically been undertaken to promote increased awareness about and response to a heart attack. For example, CDC and other federal agencies, such as the National Heart, Lung and Blood Institute and principal nonfederal partners, such as the American Heart Association, have promoted awareness of and response to heart attacks through public health messaging campaigns and improved early identification of heart attack symptoms when entering the emergency response system (*8*–*10*). Despite these promotion efforts, general knowledge about the symptoms of a heart attack remain suboptimal. Consistent messaging campaigns should be complemented with regular contact with a health care provider because screening and evaluation might lead to early intervention.

The findings in this report are subject to at least three limitations. First, because all data were self-reported, they are subject to recall and social desirability bias. Second, the questions assessing the symptoms of a heart attack were closed-ended (yes/no) and included only the correct answers and, therefore, might overestimate knowledge. Finally, NHIS includes only civilian, noninstitutionalized persons in the United States, excluding those living in nursing homes, long-term care facilities, prisons, or other comparable settings and, therefore, might not be generalizable. A strength of the study is its large size and representative sample selection.

Because of the high prevalence and significant health impact of heart attacks, awareness of the major signs and symptoms of a heart attack and the appropriate response to the event should be common knowledge among all adults. However, the suboptimal knowledge among U.S. adults identified in this study, especially among racial/ethnic minority groups, those with lower levels of education, and those with more CVD risk factors, highlight a need for enhanced and focused educational efforts. Clinical, community, and public health efforts are needed to continue to systematically improve the awareness of heart attack symptoms throughout the United States.

**Summary**

**What is already known about this topic?**

An estimated 750,000 heart attacks occur annually in the United States. Early intervention is critical in reducing morbidity and mortality. Improving public knowledge of the signs and symptoms of a heart attack can lead to improved survival and better outcomes.

**What is added by this report?**

Analysis of National Health Interview Survey data for 2008, 2014, and 2017 found that knowledge of five common signs and symptoms of a heart attack and the appropriate emergency response increased significantly (from 40% to 50% and from 92% to 95%, respectively); however, sociodemographic disparities in knowledge persist.

**What are the implications for public health practice?**

A multifaceted approach across clinical and community settings is needed to increase awareness.
